# Association between iron deposition and emotional disorder in Parkinson’s patients: a systematic review and meta-analysis

**DOI:** 10.1186/s12883-026-04866-w

**Published:** 2026-04-06

**Authors:** Kaidong Chen, Binfen Xu, Yi Ji, Wanyu Hao, Ruixuan Zhang, Liujia Lu, Yao Lu, Feng Wang, Qunfeng Tang, Li Zhang, Xiangming Fang

**Affiliations:** 1 Department of Radiology, The Affiliated Wuxi People’s Hospital of Nanjing Medical University, Wuxi, Jiangsu, 214023 China; 2 Department of Neurology, The Affiliated Wuxi People’s Hospital of Nanjing Medical University, Wuxi, Jiangsu, 214023 China

**Keywords:** Parkinson’s disease, Brain iron deposition, Anxiety, Depression, Meta-analysis

## Abstract

**Objective:**

Depression, anxiety and apathy are the most common emotional symptoms in patients with Parkinson’s disease (PD). However, the association between these symptoms and brain iron deposition remains unclear. This study aimed to systematically evaluate the strength of association between brain iron deposition and anxiety, depression, and apathy in PD patients.

**Methods:**

A systematic search was conducted in PubMed, Embase, and Web of Science databases from their inception to September 2025 to include eligible observational studies investigating the association between brain iron deposition and anxiety, depression, or apathy in PD patients. Heterogeneity among studies was assessed via Cochran’s Q test and I^2^ statistic, and a fixed-effects or random-effects model was selected to pool effect sizes based on the heterogeneity level. Subgroup analyses (stratified by outcome measures and brain regions), sensitivity analysis, and publication bias assessment (Egger’s test and Begg’s test) were conducted to verify the robustness of the results.

**Results:**

A total of 8 cross-sectional studies were finally included, with a total sample size of 855. The overall risk of bias of the included studies was manageable. Meta-analysis results showed a significant association between brain iron deposition and the occurrence of anxiety and depression in PD patients, with a pooled standardized mean difference (SMD) of 0.95 (95% confidence interval [95%CI]: 0.73–1.17). The results of the subgroup analysis showed that the results of the depression subgroup were highly stable (I^2^ = 25%). In brain region stratification, iron deposition in the substantia nigra (SMD = 1.01, 95%CI: 0.82–1.19) and general subcortical nuclei (SMD = 0.53, 95%CI: 0.23–0.84) were significantly associated with emotional symptoms, and the heterogeneity among the subgroups was low. Only 1 study was included in the apathy subgroup or the cortical subgroup.

**Conclusion:**

Brain iron deposition in PD patients is significantly associated with the occurrence of anxiety and depression, with the substantia nigra and general subcortical nuclei as the core associated brain regions. These findings can provide potential biomarkers for the early prediction and targeted intervention of emotional symptoms in PD.

## Introduction

Parkinson’s disease (PD) is a chronic progressive neurodegenerative disorder, characterized by the degeneration or loss of nigrostriatal dopaminergic neurons and the pathological deposition of Lewy bodies [1]. Due to the presence of characteristic motor impairments, including tremor, bradykinesia, rigidity, and impaired gait and posture, PD has long been widely recognized as a movement disorder [2]. Clinical research has mainly focused on the motor symptoms of PD; however, with in-depth investigations, the clinical hazards of non-motor symptoms (NMS) have gradually become prominent [3]. Among them, anxiety, depression, and apathy, as the most common emotion- and cognition-related NMS in PD patients, have a significantly higher prevalence than in the healthy population [4]. They not only directly lead to a significant reduction in patients’ quality of life scores but also exacerbate motor function decline, becoming key factors affecting the prognosis of PD patients.

The association between brain iron metabolism imbalance and neurodegenerative diseases has emerged as a research hotspot in neuroscience in recent years. A growing body of evidence indicates that iron homeostasis imbalance plays an important role in the pathogenesis of PD, which may be related to cellular damage and oxidative stress [5]. Imaging studies using techniques such as magnetic resonance imaging (MRI) have confirmed that significant iron deposition exists in the basal ganglia regions (e.g., substantia nigra and globus pallidus) of PD patients, and the degree of iron deposition is positively correlated with the severity of motor disorders [6]. On this basis, some scholars hypothesize that brain iron deposition may be involved in the occurrence and development of anxiety, depression, and apathy in PD patients by affecting the function of brain regions related to emotional regulation [7]. However, there may be varying degrees of association between iron deposition and depression, anxiety and apathy.

This study aims to integrate existing research evidence through systematic review and meta-analysis, and clarify the association between brain iron deposition quantified by quantitative susceptibility mapping (QSM) and the prevalence of anxiety, depression, and apathy in PD patients. It will provide evidence-based medical evidence for elucidating the pathogenesis of emotion-related NMS, and offer scientific references for the clinical development of targeted prevention and intervention strategies.

## Methods

### Literature search strategy

This study was conducted in accordance with the Preferred Reporting Items for Systematic Reviews and Meta-Analyses (PRISMA) principles to ensure methodological rigor [8]. The researcher systematically searched three English databases, including PubMed, Embase, and Web of Science, with the search period from database inception to September 2025. The objective was to comprehensively collect observational studies investigating the association between brain iron deposition and anxiety, depression, or apathy in patients with PD.

A search strategy combining Medical Subject Headings (MeSH) terms and free-text words was constructed, with core retrieval terms including “Parkinson Disease”, “Parkinson’s Disease”, “Iron Deposition”, “Iron Overload”, “Anxiety”, “Depression”, and “Apathy”. The search terms were adjusted according to the retrieval rules of each database to optimize search sensitivity. Additionally, the reference lists of included studies were manually searched to supplement potentially missed literature.

### Study selection

Studies were included if they met the following criteria: (1) Study design: Published observational studies, including cross-sectional studies, cohort studies, and case–control studies; (2) Study population: Patients with a definitive diagnosis of PD; (3) Exposure factor: Brain iron deposition as the core exposure variable, quantified by QSM; (4) Outcome measures: Explicit reporting of the prevalence of anxiety, depression, or apathy.

Studies were excluded based on the following criteria: (1) Non-human studies, reviews, case reports, conference abstracts, or editorial articles; (2) Lack of key data (e.g., effect size, sample size, standard deviation) that could not be obtained by contacting the corresponding authors; (3) Duplicate publications: Multiple articles from the same research team based on identical datasets.

### Data extraction

After the initial screening of titles and abstracts, full texts of potentially eligible studies were reviewed to confirm final inclusion. Extract the following data: (1) Publication characteristics: Author and publication year; (2) Demographic: Sample size of each group, source; (3) Emotional symptom data: Assessment tools; prevalence; (4) Location of brain iron deposition.

### Quality assessment

The risk of bias assessment tool (Risk of Bias 2.0, ROB 2.0) recommended by the Cochrane Collaboration was adopted, with assessment dimensions including random sequence generation, allocation concealment, blinding of participants and researchers, blinding of outcome assessment, incomplete outcome data, and selective reporting.

### Statistical analysis

Heterogeneity among studies was first evaluated using the Cochran’s Q test and I^2^ statistic, with a statistical significance threshold set at *P* < 0.05. A fixed-effects model was used to pool effect sizes if no significant heterogeneity existed (*P* > 0.05 and I^2^ < 50%); otherwise, a random-effects model was applied to account for between-study heterogeneity.

The results have been reported as standardized mean differences (SMDs) and as 95% confidence intervals (95% CI). Subgroup analyses were conducted by outcome type (anxiety, depression, apathy) and brain region (substantia nigra, general subcortical nuclei, cortical region). Sensitivity analysis was performed by sequentially excluding each included study to assess the robustness of the pooled results. Publication bias was evaluated using funnel plot symmetry visual inspection, Begg’s test, and Egger’s linear regression test, with *P* > 0.05 indicating no significant publication bias.

## Results

### Study selection and characteristics

**Fig. 1:**
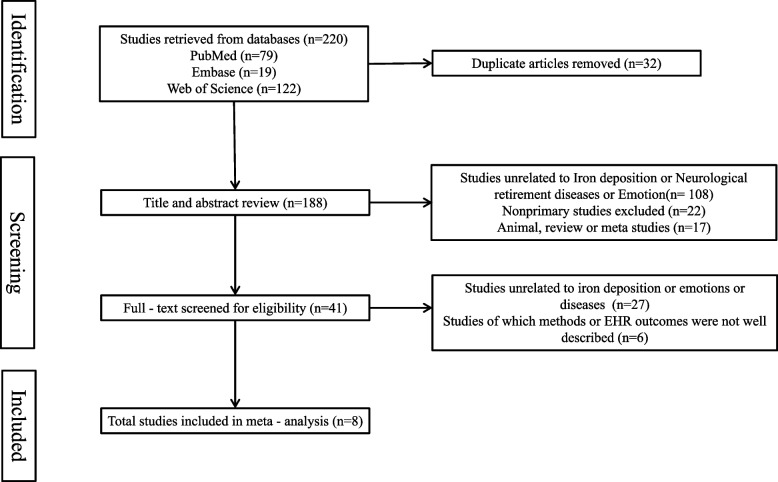
Flow diagram of study selection

The initial literature search retrieved 220 articles, among which 32 were duplicates. After sequential screening of titles, abstracts, and full texts, 8 studies meeting the inclusion criteria were finally included [9–16]. The detailed screening process is shown in Fig. 1. The total sample size of the included studies was 855, consisting of 484 participants in the experimental group and 371 in the control group (healthy controls). Main characteristics of the included studies are summarized in Table 1, with sample sizes ranging from 42 to 285. Regarding publication time, studies published in 2025 accounted for the highest proportion, and 87.5% of the studies were published in the past five years, indicating high timeliness.

**Table 1:** Characteristics of the included studies

Author	Data collection	Region	Symptoms	Encephalic region	Sample (Total)	Sample (Healthy Control)	Sample (Experiment)	Measurement of iron deposition	Clinical scale	PD staging	Findings
Li Zhang et al	2025	China	Apathetic	SNc	61	32	29	QSM	AS	H-Y stage 2.10 ± 0.74, early- to mid-stage PD	PD with pure apathy exhibited more extensive increased iron deposition in SFGmed
George Edward Calver Thomas et al	2020	Britain	Anxiety	Subcortical Nuclei	137	37	100	QSM	HADS	Early- to mid-stage PD; within 10 years of diagnosis	QSM increases in PD. The anxiety score of PD patients was significantly higher than that of the control group (5.94 vs. 3.97, *P* < 0.05).
Jiaqi Wen et al	2025	China	Depression	Dorsomedial Functional Subregion of the Substantia Nigra	285	120	165	QSM	HAMD	H-Y stage 2.10 vs 2.60	HAMD scores were higher in the PD group than in the HC group (*p* < 0.001). The VBA_Limbic SN QSM values were positively correlated with and HAMD score (*r* = 0.190, *p* = 0.016/*p*_FDR_ = 0.036).
Weihang Guo et al	2024	China	Depression	SNc	75	35	40	QSM	HAMD	Median disease duration 3 years	The iron level in the SNc is positively correlated with the HAMD scores (*r* = 0.313, *P* = 0.049).
Ying Yan et al	2023	China	Anxiety	Subcortical Nuclei	60	36	24	QSM	BAI	Median disease duration 58 months	The iron deposition in the bilateral substantia nigra of patients with PD was higher than that of the HC group. the BAI and BDI scores in the PD group were higher than those in the HC group (*P* < 0.05).
Kaidong Chen et al	2023	China	Anxiety	Medial prefrontal cortex	42	26	16	QSM	HAMA	H-Y stage 1–3	Significant positive correlations were found between the HAMA scores and the mean QSM values of the cluster located on the left mPFC (*r* = 0.630, *P* < 0.01) and the cluster located on the right mPFC (*r* = 0.519, *P* = 0.039) in the single PD-A group.
Hedi An et al	2018	China	Depression/Anxiety	Bilateral Substantia Nigra	75	31	44	QSM	HAMA	H-Y ≤ 2.5 (mild); H-Y ≥ 3 (advanced)	The iron content in substantia nigra was significantly correlated with Montgomery Asberg Depression Rating Scale and Hamilton Anxiety Scale scores in both mild symptom severity (*r* = 0.429, *P* = 0.013; *r* = 0.432, *P* = 0.004) and advanced symptom severity (*r* = 0.758, *P* = 0.007; *r* = 0.683, *P* = 0.020).
Yongyan Fan et al	2024	China	Depression	Bilateral Substantia Nigra	120	54	66	QSM	HAMD	H-Y stage 1–2.5 vs H-Y stage 3–5	There was a weak but significant positive correlation between plasma lipocalin-2 levels and HAMD scores (*r* = 0.273, *P* = 0.027)

*SNc* Substantia nigra pars compacta, *PD* Parkinson’s disease, *QSM* Quantitative susceptibility mapping, *AS* Apathy scale, *HADS* Hospital anxiety and depression scale, *HAMD* Hamilton depression rating scale, *BAI* Beck anxiety inventory, *HAMA* Hamilton anxiety rating scale, *H-Y* Hoehn–Yahr staging

### Quality assessment

**Fig. 2:**
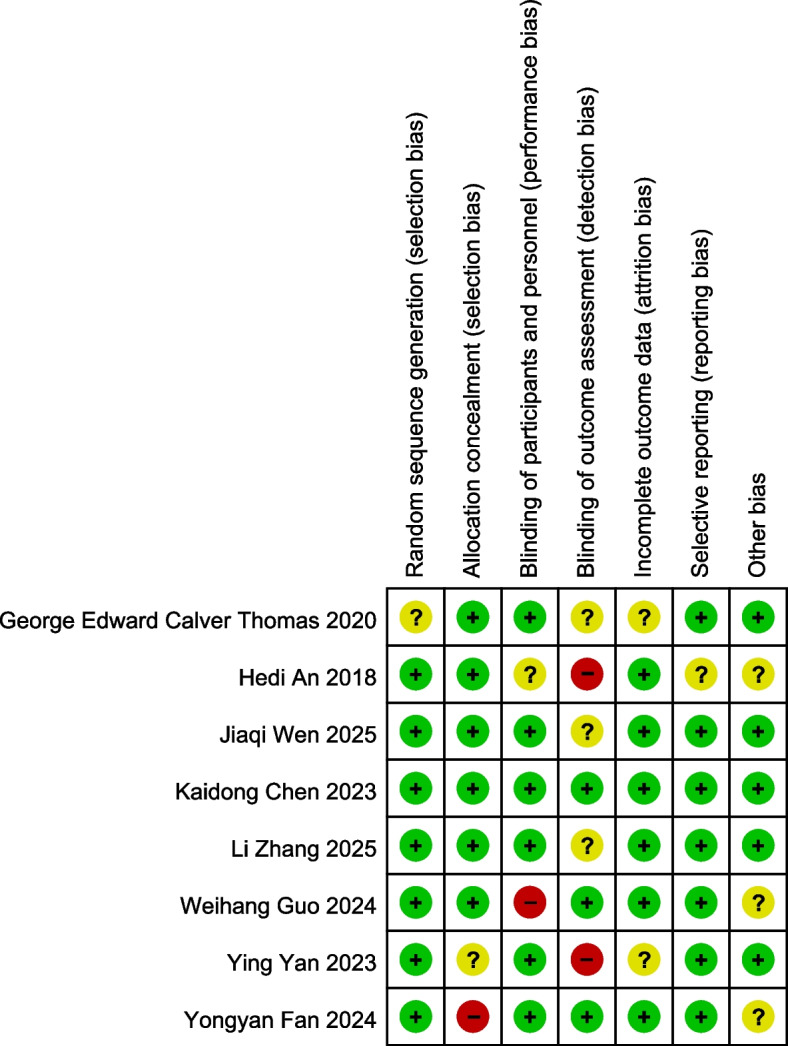
Risk of bias assessment plot of included studies

The quality evaluation results showed that most studies had a low risk of bias in terms of random sequence generation, incomplete outcome data, and selective reporting. Some studies had an “unclear risk” in allocation concealment and blinding of participants and researchers, but no study showed a high risk of bias (Fig. 2). Overall, the risk of bias of the included studies was manageable without systematic bias, providing a solid methodological basis for the authenticity and reliability of the pooled effect sizes in the meta-analysis.

### Meta-analysis of emotional symptoms and iron deposition in PD patients

A total of 8 studies focusing on the association between brain iron deposition and the prevalence of anxiety, depression, and apathy in PD patients were included, with a total sample size of 855. Heterogeneity analysis revealed heterogeneity among the included studies (I^2^ = 51%), so a random-effects model was used to pool the effect sizes.

**Fig. 3:**
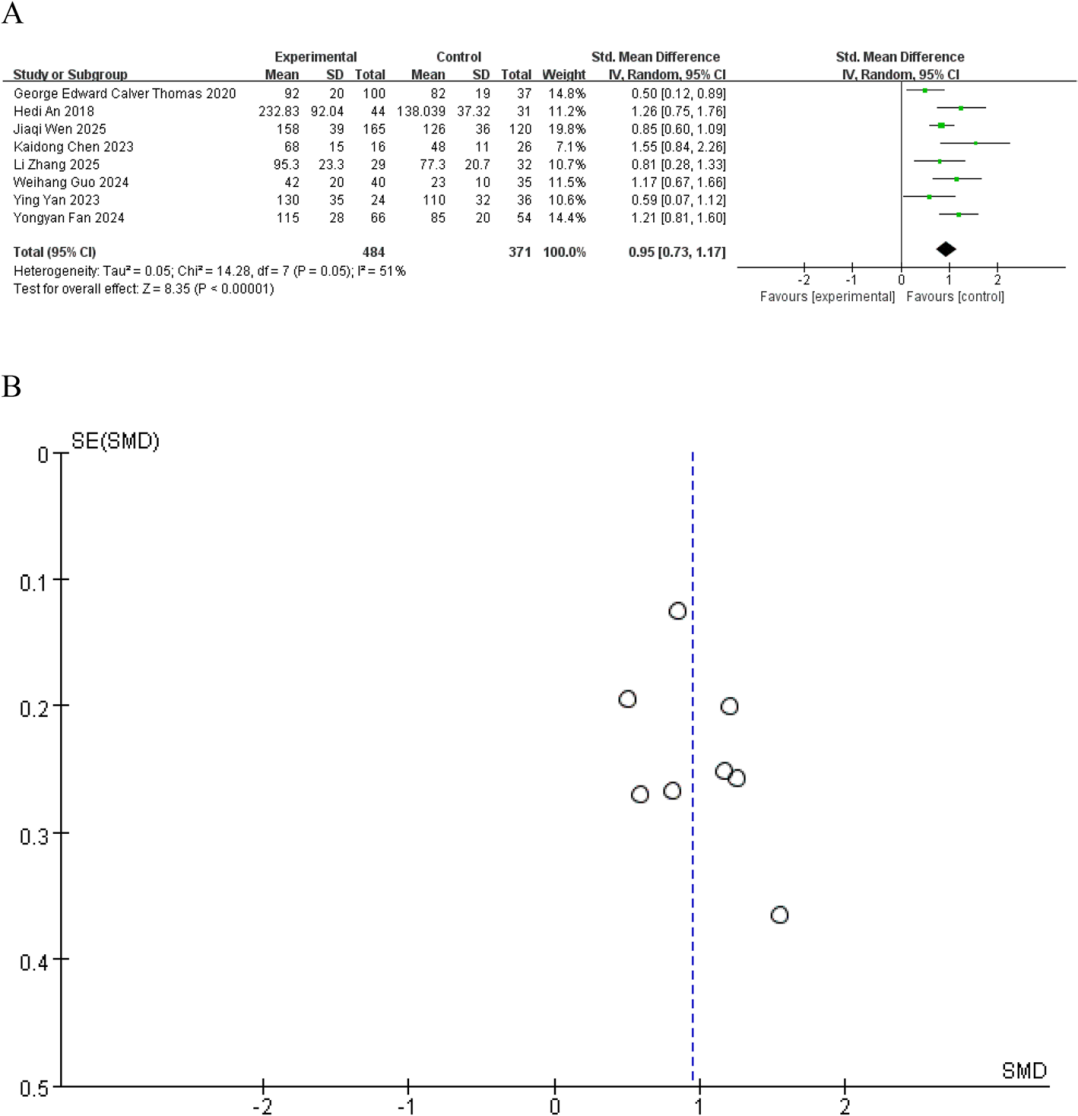
Forest plot (**A**) of the overall effect of brain iron deposition on emotional symptoms in PD patients and funnel plot for publication bias assessment (**B**)

The results of the overall effect showed that the SMD was 0.95 (95%CI: 0.73–1.17, Z = 8.35, *P* < 0.00001), indicating that the effect of the experimental group was significantly higher than that of the control group, with a statistically significant difference (Fig. 3A). Publication bias was evaluated using a funnel plot, which showed symmetric distribution of data points, suggesting no significant publication bias (Fig. 3B).

### Subgroup analyses

**Fig. 4:**
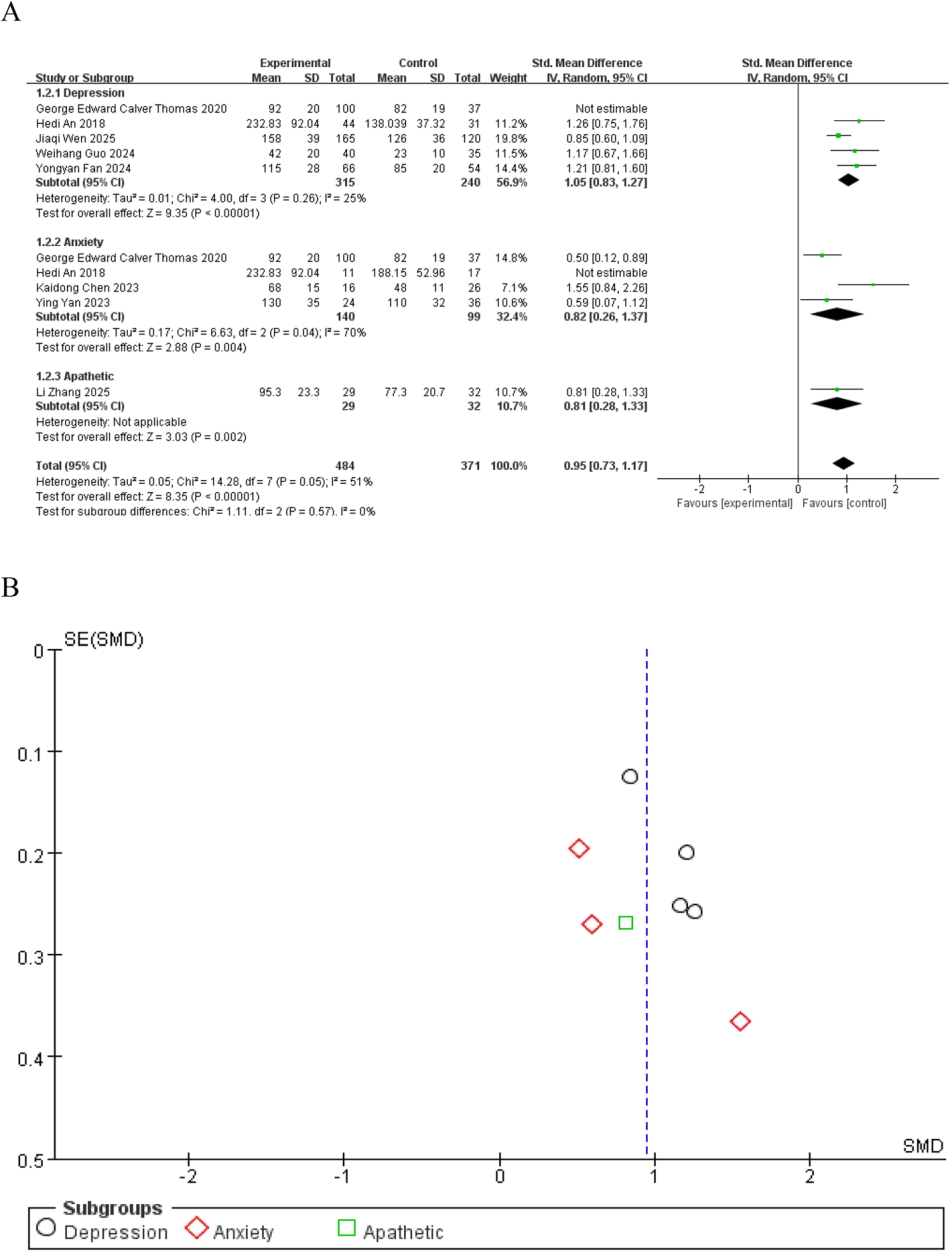
Subgroup analysis forest plot (**A**) stratified by emotional symptoms and funnel plot for publication bias assessment (**B**)

Studies were stratified into subgroups by outcome measures (depression, anxiety, apathy), and analyses were performed using a random-effects model (Fig. 4A). The depression subgroup included 5 cross-sectional studies with low heterogeneity (I^2^ = 25%). The pooled SMD was 1.05 (95%CI: 0.83–1.27), and the overall effect test showed Z = 9.35 (*P* < 0.00001), indicating a significant association between iron deposition and depression in PD patients. The anxiety subgroup included 4 cross-sectional studies, with a pooled SMD of 0.82 (95%CI: 0.26–1.37) and an overall effect test Z = 2.88 (*P* = 0.004), suggesting a significant association between iron deposition and anxiety in PD patients. Only 1 study was included in the apathy subgroup, so heterogeneity could not be evaluated. The funnel plot indicates that there is no significant publication bias (Fig. 4B).

**Fig. 5:**
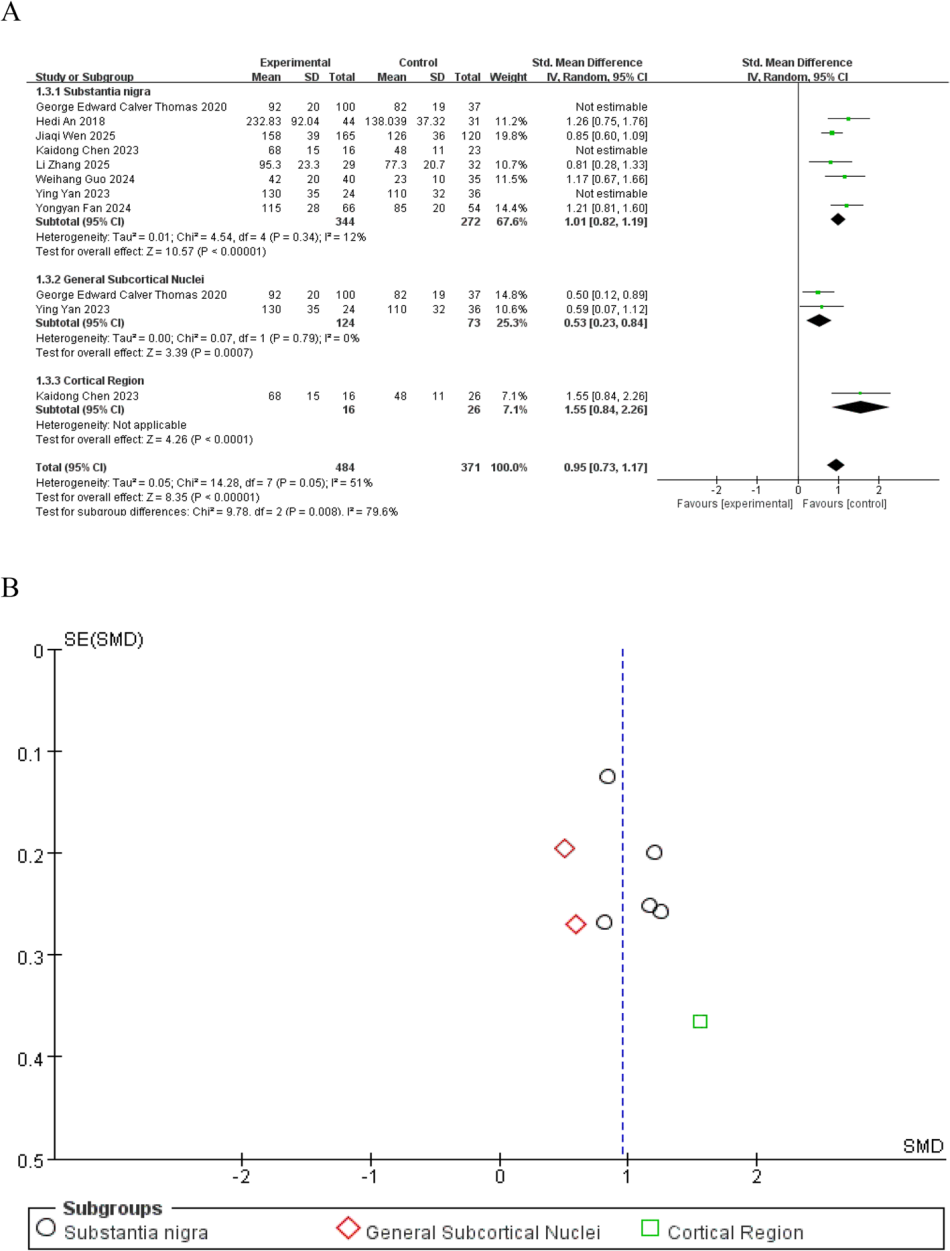
Subgroup analysis forest plot (**A**) stratified by brain regions and funnel plot for publication bias assessment (**B**)

Further subgroup analysis was conducted according to the brain region of iron deposition detection (substantia nigra, general subcortical nuclei, and cortical regions) (Fig. 5A). The substantia nigra subgroup had a pooled SMD of 1.01 (95%CI: 0.82–1.19) with heterogeneity I^2^ = 12% (*P* = 0.34), and the overall effect test Z = 10.57 (*P* < 0.00001). The general subcortical nuclei subgroup had a pooled SMD of 0.53 (95%CI: 0.23–0.84), and the overall effect test Z = 3.39 (*P* = 0.0007). Only 1 study was included in the cortical region subgroup. The overall pooled SMD was 0.95 (95%CI: 0.73–1.17) with heterogeneity I^2^ = 79.6% (*P* = 0.008), and the overall effect test Z = 8.35 (*P* < 0.00001). Publication bias evaluation showed no significant bias (Fig. 5B).

### Sensitivity analysis

**Fig. 6:**
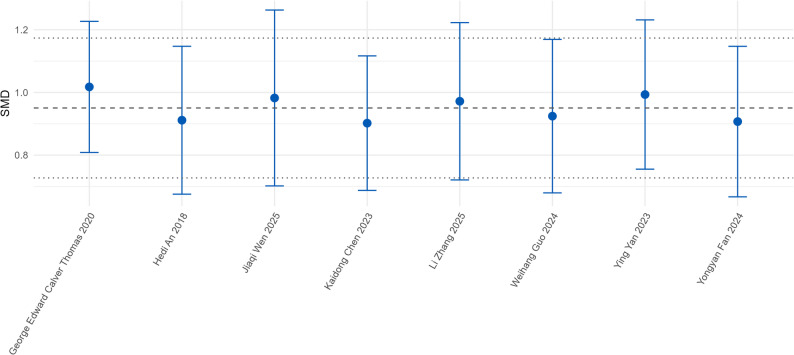
Sensitivity analysis plot by sequentially excluding each included study

Sensitivity analysis was performed by sequentially excluding each included study. The results showed that after excluding any single study, the fluctuation range of the pooled SMD was 0.72–1.17, which was highly consistent with the overall SMD (0.95, 95%CI: 0.73–1.17) (Fig. 6). Further observation revealed that the 95%CI of the pooled effect size did not cross the null line after excluding any study, and there was no abnormal increase or decrease in study heterogeneity. These results indicate that the individual effect of the included studies on the overall pooled result is small, and the pooled effect size of this meta-analysis has good robustness.

### Publication bias assessment

Egger’s linear regression test and Begg’s rank correlation test were used to jointly assess publication bias. Egger’s test showed t = 0.9994, df = 6, *P* = 0.3562, with a limit estimate of 0.60 (95%CI: −0.24–1.43). Begg’s test showed z =0.74, *P* = 0.4579. The *P*-values of both tests were greater than 0.05, suggesting no significant asymmetry in the funnel plot of this meta-analysis and no obvious publication bias. This result indicates that the included 8 studies have a low risk of publication bias, further supporting the reliability and authenticity of the pooled effect size.

## Discussion

This study systematically retrieved and integrated data from 8 eligible observational studies to comprehensively evaluate the association between brain iron deposition and emotional symptoms (anxiety, depression, and apathy) in patients with PD. The research provides the latest evidence-based medical evidence for elucidating the underlying pathophysiological mechanisms.

This study found that there is a significant positive correlation between brain iron deposition and the occurrence of depression in patients with PD. This conclusion is highly consistent with the findings of multiple studies. A study by Wen et al. revealed a significant positive correlation between VBA_Limbic substantia nigra (SN) QSM values and Hamilton Depression Scale (HAMD) scores [14]. Francesco et al. found that patients with depression have higher brain iron content, supporting previous studies suggesting a link between iron homeostasis and depression [17, 18]. Guo et al. (2024) found a significant positive correlation between iron levels in the substantia nigra pars compacta (SNc) and HAMD scores (*r* = 0.313, *P* = 0.049) [12], which aligns with the pooled effect of this meta-analysis. As the core structure of the extrapyramidal system, the basal ganglia not only regulate motor function but also participate in emotional modulation through the prefrontal-basal ganglia circuit and limbic-basal ganglia circuit, which has been well-documented [19]. Experiments on Nrf2-regulated iron homeostasis have shown that iron overload exacerbates chronic unpredictable mild stress (CUMS)-induced depressive-like behaviors, accompanied by impaired hippocampal synaptic function [20]. And the iron overload may induce emotional abnormalities by accelerating dopaminergic neuron apoptosis [21].

Similarly, cerebral iron deposition is significantly associated with the occurrence of anxiety in PD patients. Based on recent QSM studies, the Hamilton Anxiety Scale (HAMA) scores of PD patients are positively correlated with iron content in “fear circuit” regions such as the medial prefrontal cortex, anterior cingulate cortex, and hippocampus, and the QSM values in these regions increase linearly with the severity of anxiety [10]. The mechanism of iron deposition-induced anxiety is closely related to the abnormal activation of the fear circuit and the impairment of hippocampal regulatory function. A decrease in the control ability of the hippocampus leads to the recovery of previously extinguished fear conditioning, which is considered one of the mechanisms underlying the development of anxiety disorders [22].

Although the study indicated that iron deposition levels in the left medial superior frontal gyrus (SFGmed), anterior cingulate cortex (ACC), thalamus, and superior temporal gyrus are positively correlated with Apathy Scale (AS) scores [16], only 1 study was included in the apathy subgroup (SMD = 0.81, *P* = 0.002). However, this result should be interpreted with caution, which may be attributed to two factors: on the one hand, the limited number of included studies and relatively small sample size restrict the generalizability of the findings; on the other hand, apathy has a specific pathological mechanism. Apathy is believed to be mainly associated with the disruption of anterior cingulate cortex-ventral striatum connectivity, and its neurotransmitter basis relies more on the motivational circuit of the dopaminergic system rather than simple iron metabolism abnormalities [23]. The specificity of this pathological mechanism may weaken the direct impact of iron deposition on apathy, and future studies with larger sample sizes are needed to further verify this potential association.

In PD, the degeneration of substantia nigra dopaminergic neurons is often accompanied by increased local iron load [24]. Studies have pointed out that abnormal iron accumulation first occurs in the substantia nigra, red nucleus, and basal ganglia (especially the putamen and globus pallidus), and iron deposition in specific nuclei is directly associated with emotional disorders [25]. Early research reported that lesions in the left posterior globus pallidus are significantly correlated with secondary depression, suggesting that globus pallidus dysfunction can cause emotional disturbances [26]; another study on PD patients with type 2 diabetes noted that diabetes can exacerbate iron deposition in the right red nucleus (RN) and increase anxiety levels [27]. In contrast, the lack of a significant association between cortical iron deposition and emotional symptoms suggests that the cerebral cortex is not a core target of iron deposition in influencing PD-related emotional symptoms, providing a clear direction for future studies to focus on the basal ganglia-limbic system. Notably, significant between-study heterogeneity was detected in the brain region-stratified subgroup analysis (I^2^ = 79.6%). The high level of heterogeneity may be due to the clinical differences among the studies, including different Parkinson’s disease Hoehn-Yahr stages, disease duration, the use of dopaminergic drugs, as well as the differences in the scales used to evaluate the results and the brain regions targeted in different studies. This could lead to differences in the measurement results of iron deposition and the assessment results of emotional symptoms; therefore, the interpretation of the combined effect size should be carried out with caution, and future studies should adopt unified clinical standards and standardized assessment protocols to reduce this heterogeneity.

The results of this study are highly consistent with the conclusions of most recent observational studies. For example, another included study confirmed that PD patients had increased QSM values and higher anxiety scores compared with healthy controls [13]. Unlike previous small-sample studies, this meta-analysis integrated data from 855 participants, significantly improving statistical power. In particular, subgroup analyses clarified the differential associations across different brain regions, making up for the limitations of single-center studies in representativeness. Furthermore, this study is the first to include three core emotional symptoms (anxiety, depression, and apathy) in a unified analytical framework, identifying the symptom-specificity of iron deposition associations. It also refined brain region-stratified analyses, confirming the core role of the substantia nigra and general subcortical nuclei, and providing more precise targets for further research.

The findings of this study suggest that iron deposition in the substantia nigra and general subcortical nuclei can serve as potential predictive biomarkers for anxiety and depression in PD patients. Clinically, routine imaging detection of iron deposition can be performed in PD patients, and early psychological screening and intervention can be initiated for high-risk populations. As a non-invasive quantitative MRI technique, QSM protocol can be easily integrated into routine brain MRI examinations for Parkinson’s disease patients without requiring additional contrast agents, demonstrating strong clinical feasibility and applicability. Routine QSM-based iron deposition assessment can assist clinicians in early identification of PD patients at high risk for mood disorders, enabling early warning stratification management of non-motor symptoms and thereby optimizing individualized clinical decision-making. Iron metabolism regulation may represent a novel therapeutic strategy for PD-related emotional symptoms, and future clinical intervention trials are warranted. However, this study has several limitations. Firstly, all 8 included studies were cross-sectional, only confirming an association rather than a causal relationship; and the included studies adopted various clinical rating scales to assess the symptoms or signs of emotional disorders, rather than formal psychiatric diagnoses, which may introduce certain assessment bias. Secondly, there was an imbalance in subgroup sample sizes (e.g., only 1 study in the apathy subgroup). Thirdly, there is a potential risk of publication bias—although Egger’s and Begg’s tests did not indicate significant publication bias, all included studies were English-language publications with positive results, which may lead to “negative result publication bias”. Finally, there are unadjusted confounding factors, such as disease itself, PD disease duration, medication use, and comorbidities. This study was unable to conduct further stratified analyses due to limited raw data, which may overestimate the association strength.

## Conclusion

This study clarifies that brain iron deposition in PD patients is significantly correlated with the occurrence of anxiety and depression. Brain region-stratified analyses further verify that the substantia nigra and general subcortical nuclei are the core brain regions through which iron deposition influences PD-related emotional symptoms. These results offer important references for target-specific research and clinical assessment of PD-related emotional symptoms.

## Data Availability

The researcher systematically searched three English databases, including PubMed, Embase, and Web of Science.
